# Traumatic Brain Injury and Risk of Amyotrophic Lateral Sclerosis

**DOI:** 10.1001/jamanetworkopen.2025.35119

**Published:** 2025-10-02

**Authors:** Xingxing Zhu, Emma R. Russell, Donald M. Lyall, Frederick Ho, Daniel F. Mackay, Jill P. Pell, William Stewart

**Affiliations:** 1 School of Health and Wellbeing, University of Glasgow, Glasgow, United Kingdom; 2 School of Psychology and Neuroscience, University of Glasgow, Glasgow, United Kingdom; 3 Department of Neuropathology, Queen Elizabeth University Hospital, Glasgow, United Kingdom

## Abstract

**Importance:**

History of traumatic brain injury (TBI) or repetitive head impacts is associated with an increased risk of neurodegenerative disease. This association has attracted attention in recent years through the relationship between contact sports participation and the increased risk of a number of neurodegenerative diseases, including motor neuron disease or amyotrophic lateral sclerosis (ALS). However, to date, the association between TBI in the community and ALS risk remains uncertain.

**Objective:**

To leverage population-level health records to explore the association between a history of TBI and subsequent ALS risk.

**Design, Setting, and Participants:**

This retrospective cohort study accessed UK-wide electronic health record (EHR) data from individuals 18 years or older with TBI history and age-, sex-, and area deprivation–matched general population comparators. EHR data were available from January 1, 2005, to December 31, 2020, with database interrogation performed on February 11, 2021, and data analysis conducted between June 1, 2023, and October 3, 2024.

**Exposure:**

Documented history of TBI.

**Main Outcomes and Measures:**

Outcomes were obtained by individual-level linkage to EHR data available via Clinical Practice Research Datalink. Risk of ALS was evaluated using Cox proportional hazards regression models to investigate its association with TBI.

**Results:**

Overall, 85 690 adults with a history of TBI and 257 070 matched adults with no history of TBI were included, for a total of 342 760 participants (50.1% male; mean [SD] age, 50.7 [17.6] years). During a median 5.72 (IQR, 3.07-8.82) years of follow-up, providing 2.13 million person-years of follow-up, 150 incident ALS cases were recorded, resulting in 7.05 cases per 100 000 person-years. Risk of ALS was higher among individuals with a history of TBI compared with individuals without a TBI history (hazard ratio [HR], 2.61; 95% CI, 1.88-3.63). However, this association was time dependent, with risk confined to the 2 years following TBI (HR, 6.18; 95% CI, 3.47-11.00), but not thereafter.

**Conclusions and Relevance:**

In this retrospective cohort study of 342 760 adults, an association between TBI and subsequent risk of ALS was identified. However, this association was confined to the 2 years immediately following injury. As such, the association between TBI and higher ALS risk may indicate reverse causality, with TBI in some individuals perhaps reflecting a consequence of early, subclinical ALS.

## Key Points

**Question:**

Is there an association between traumatic brain injury (TBI) and subsequent risk of amyotrophic lateral sclerosis (ALS)?

**Findings:**

In this cohort study of 85 690 individuals 18 years or older with documented TBI and 257 070 age-, sex-, and area deprivation–matched comparators from the general population, risk of ALS was higher following TBI. However, the risk was time dependent, being highest in the 2 years immediately following TBI and attenuating thereafter to no increased risk compared with general population.

**Meaning:**

These findings suggest that increased ALS risk following TBI may reflect reverse causality, with TBI in some individuals perhaps reflecting an early consequence of subclinical ALS.

## Teaser

This cohort study leverages UK-wide electronic health records to test the hypothesis that the risk of amyotrophic lateral sclerosis is higher among individuals following traumatic brain injury compared with age-, sex-, and area deprivation–matched individuals without injury.

## Introduction

Traumatic brain injury (TBI) and repetitive head impacts (RHI) have emerged as important risk factors for the development of a range of neurodegenerative diseases.^1^ However, while increased risk of dementia and related disorders is frequently reported in association with both sport- and nonsport-related TBI, the association between TBI and the risk of amyotrophic lateral sclerosis (ALS) is less consistently reported, perhaps reflecting limitations in methodological approaches and, often, relatively small case numbers studied.^2^

Despite first suggestions of an association between TBI and ALS emerging in case reports dating to the early part of the last century,^3^ the contribution of TBI to ALS risk remains uncertain.^2^ A recent systematic review and meta-analysis identified 14 eligible studies for inclusion, with considerable variation in study design and case numbers ranging 1 to 4004.^4^ Nevertheless, this meta-analysis estimated an approximately 40% increase in ALS risk post TBI.^4^ Of note, the data suggest that risk may be highest in the years immediately following injury, raising the potential that the association might reflect reverse causality; this possibility was first suggested as early as 1911 but remains unconfirmed.^3^

Electronic health and death certification records offer the opportunity to access clinical data to conduct population level epidemiological analyses at scale. In this context, we leveraged UK-wide electronic health records (EHR) to test the hypothesis that ALS risk is higher among individuals following TBI than among age-, sex-, and area deprivation–matched comparators. Further, we assessed whether any observed ALS risk might be time dependent, being highest in the years immediately following TBI and diminishing thereafter.

## Methods

The protocol for this cohort study was approved by the Independent Scientific Advisory Committee of the Clinical Practice Research Datalink (CPRD). CPRD is an approved research resource collecting anonymized patient data from a network of primary care surgeries across the UK.^5^ Participants can opt out of their patient information being shared for research. Written informed consent from participants was not required owing to pseudonymized data. The analysis and reporting of this study are consistent with the Strengthening the Reporting of Observational Studies in Epidemiology (STROBE) reporting guideline.

### Data Sources

We conducted a population-based matched cohort study using the CPRD GOLD and Aurum datasets, which contain anonymized EHR for more than 50 million patients across 1999 UK general practices. Clinical information such as medical diagnoses, procedures, and prescriptions is recorded in both the GOLD and Aurum datasets. These CPRD datasets also have linkage to Hospital Episode Statistics (HES), which covers dates, diagnoses, and procedures relating to hospital admissions; Office for National Statistics (ONS) Mortality Data, which covers cause and date of death; and the index of multiple deprivation, which measures area-level socioeconomic status. HES and ONS linkage were only available for a subset of individuals in CPRD (39.97% in the GOLD dataset and 91.93% in the Aurum dataset).

### Study Cohort

The study cohort consisted of individuals 18 years or older at the date of the first recorded TBI event between January 1, 2005, and December 31, 2020, and their matched general population comparators. Participants with a TBI were identified in CPRD, HES and ONS datasets using disease codes specific to each dataset (eTables 1 and 2 in Supplement 1).

Each individual with TBI was matched with 3 general population comparators with no history of TBI using the calipmatch command in Stata, version 17 (StataCorp LLC), with caliper matching for year of birth (within 3 years) and exact matching for sex and registered general practice as a proxy for area deprivation. The index date for the study was defined as the date of the first recorded TBI event. Follow-up of comparators began on the same day as their respective match individual with a history of TBI. Incident ALS diagnoses were identified in CPRD, HES, and ONS using appropriate CPRD codes (eTable 3 in Supplement 1) or *International Statistical Classification of Diseases, Tenth Revision* code G12.2, with the date of diagnosis defined as the date of first coding of ALS from any source. Further details regarding eligibility and data management are provided in the eMethods, eTable 4, and eTable 5 in Supplement 1. All analyses included data up to December 31, 2020, with database interrogation performed on February 11, 2021.

### Statistical Analysis

Data were analyzed from June 1, 2023, to October 3, 2024. Cox proportional hazards regression models were used to estimate the association between TBI and ALS, with the result expressed as hazard ratios (HRs) and related 95% CIs. The proportional hazards assumption was tested using scaled Schoenfeld residuals. Where the assumption of proportional hazards did not hold, a time-varying model was used to derive HRs over intervals of follow-up. All tests were conducted using Stata, version 17, and were 2 sided, with the significance level set a priori at *P* < .05.

## Results

### Demographic Characteristics

In total, 85 690 individuals 18 years or older with a history of TBI and 257 070 matched general population comparators (eFigures 1 and 2 in Supplement 1) were followed up for a median of 5.72 (IQR, 3.07-8.82) years (range, 1 day to 16 years), providing 2.13 million person-years of follow-up. Of the total 342 760 participants, 170 984 (49.9%) were female and 171 776 (50.1%) were male; mean (SD) age at index date was 50.7 (17.6) years. Ethnicity data were available in 267 810 individuals, of whom 243 705 (91.0%) were White. Full age, sex, and practice data were available for all participants. The mean (SD) age at the start of follow-up was 50.8 (17.7) years for TBI cases and 50.7 (17.6) years for non-TBI comparators. Reflecting the matching process, there were no differences in demographic characteristics between individuals with TBI and their general population comparators (Table 1).

**Table 1.** Demographic Characteristics of the Study Population

Characteristic	TBI cohort (n = 85 690)	Comparators (n = 257 070)	*P* value
Follow-up, median (IQR), y	5.6 (3.0-8.8)	5.8 (3.1-8.8)	<.001^a^
Age at index date, mean (SD), y	50.8 (17.7)	50.7 (17.6)	.33^b^
Sex, No. (%)			
Female	42 746 (49.9)	128 238 (49.9)	Matched
Male	42 944 (50.1)	128 832 (50.1)	
Quintiles of IMD, No. (%)			
1 (Least deprived)	14 489 (16.9)	43 467 (16.9)	Matched
2	13 456 (15.7)	40 368 (15.7)	
3	15 375 (17.9)	46 125 (17.9)	
4	20 205 (23.6)	60 615 (23.6)	
5 (Most deprived)	22 165 (25.9)	66 495 (25.9)	

Abbreviations: IMD, index of multiple deprivation; TBI, traumatic brain injury.

^a^ Calculated using the Mann-Whitney test.

^b^ Calculated using the independent samples *t* test.

### Risk of ALS 

In total, 150 ALS diagnoses were captured during follow-up, reflecting an ALS incidence in the entire study population of 7.05 (95% CI, 6.00-8.27) per 100 000 person-years, with risk higher among individuals with TBI than among their matched general population comparators. Thus, over follow-up, incident ALS diagnoses were recorded for 69 of 85 690 individuals with TBI (0.08%) and 81 of 257 070 matched general population comparators (0.03%) (HR, 2.61; 95% CI, 1.88-3.63; *P* < .001) (Table 2), reflecting ALS incidences of 13.12 (95% CI, 10.36-16.61) and 5.05 (95% CI, 4.06-6.28) per 100 000 person-years, respectively. However, the proportional hazards assumption was not met, suggesting time-dependent variability in risk. This was confirmed in a time-dependent analysis, which revealed risk of ALS was highest in the 2 years immediately following TBI (HR, 6.18; 95% CI, 3.47-11.00; *P* < .001), attenuating thereafter to no different than that of matched population comparators (Table 2 and Figure). There was no difference in mean (SD) age at first recording of an ALS diagnosis (69.7 [11.1] vs 68.2 [11.6] years; *P* = .41) or in age at death with ALS (72.2 [10.8] vs 72.0 [10.3] years; *P* = .91) between individuals with TBI and their matched general population comparators.

**Table 2.** Time-Dependent Risk of Developing ALS Following TBI

Variable	No. of observed ALS cases		HR (95%CI)	*P* value^a^
	TBI cohort	Comparators		
Overall	69	81	2.61 (1.88-3.63)	<.001
Time from TBI to ALS diagnosis				
0 to ≤2 y	36	18	6.18 (3.47-11.00)	<.001
>2 to ≤4 y	9	21	1.43 (0.64-3.19)	.38
>4 to ≤6 y	11	16	1.80 (0.83-3.91)	.14
>6 to ≤8 y	7	7	2.48 (0.86-7.15)	.09
>8 to ≤16 y	6	19	1.04 (0.40-2.69)	.94

Abbreviations: ALS, amyotrophic lateral sclerosis; HR, hazard ratio; TBI, traumatic brain injury.

^a^ Calculated using Cox proportional hazards regression.

**Figure.**
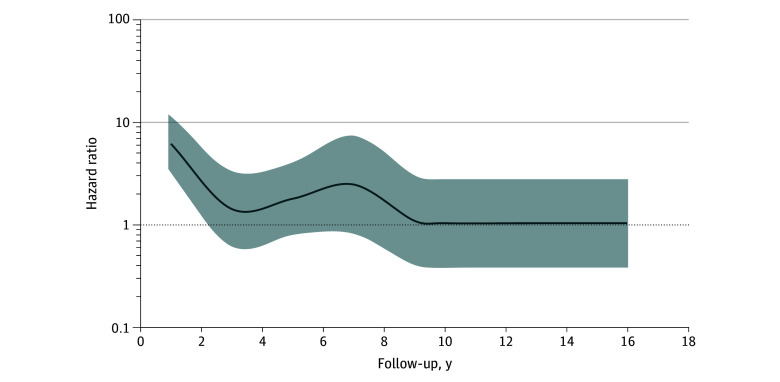
Hazard Ratios of Developing Amyotrophic Lateral Sclerosis Following Traumatic Brain Injury The line indicates the hazard ratios; the shading represents the 95% CIs.

## Discussion

In this general population cohort study accessing UK-wide EHR data, a history of TBI was associated with approximately 2.6-fold higher risk of a subsequent ALS diagnosis when compared with matched individuals from the general population. However, this association appeared time dependent, with higher risk of ALS restricted to the 2 years immediately following TBI and attenuating thereafter. Finally, we observed no difference in age at diagnosis or at death due to ALS in individuals with TBI compared with matched individuals from the general population.

Several studies exploring ALS^6,7,8^ have documented the time interval between manifestation of first symptoms and confirmation of an ALS diagnosis, with reported median times ranging from approximately 9 months to more than 2 years. In this context, it seems plausible that the high ALS risk we observe in the years immediately following TBI might represent reverse causality. In other words, rather than TBI precipitating a neurodegenerative process culminating in ALS, TBI might represent an early complication of disease in individuals with preclinical ALS at risk of falls or other events culminating in TBI. Thereafter, in the months or years following TBI, the diagnosis of ALS emerges, consistent with the typical timeline to secure a diagnosis from first symptom presentation. Diagnostic coding datasets capture information on established disease at the time of a clinical diagnosis and, typically, do not hold information on prodromal symptoms or signs in the period prior to clinical diagnosis that might herald evolving disease. Clearly, to explore the potential reverse causality raised by our data, information regarding any signs or symptoms heralding ALS onset in the period prior to presentation with TBI would be informative.

### Limitations

As our dataset was extracted from diagnostic coding of outcomes captured in EHR, this study has inherent limitations. Not all individuals with TBI will seek medical attention, with individuals among our non-TBI comparators inevitably having unrecorded TBI. In support of this, analysis of UK Biobank data shows relatively poor overlap between TBI coded from the *International Statistical Classification of Disease, Tenth Revision*, compared with self-reported head injury.^9^ In part, this will be influenced by injury severity, with the result that patients identified with TBI in our study will be biased toward more moderate or severe injury. Going forward, comprehensive, prospective data collection capturing lifetime TBI exposures across all severities of injury will be required to pursue this intriguing association between TBI and ALS, among other lifelong health outcomes. Moreover, while we saw no increased ALS risk beyond 2 years of follow-up post TBI, we cannot exclude the possibility of a delayed increase in risk at time points beyond 16 years. Unfortunately, limitations of data availability in CPRD are such that we were unable to extend beyond 16 years of follow-up in this study. Future study designs extending to later time points should be considered, should data availability be supportive. A further limitation in our dataset surrounds availability of granular data on wider known or suspected ALS risk factors. By example, information on ethnicity was not available for more than 20% of our study population. As a result, reflective of the UK demographic characteristics and the number of incident ALS cases identified, our final dataset contained less than 5 individuals with ALS of minority ethnicity, precluding any meaningful exploration of the potential influence of ethnicity on our observations.

## Conclusions

In this UK-wide, general population cohort study of 85 690 individuals with TBI compared with 257 070 age-, sex-, and area socioeconomic status–matched comparators, we identified an approximately 2.6-fold increased risk of ALS following TBI. However, this higher risk was restricted to the first 2 years immediately following TBI and diminished thereafter. Further investigation should explore the potential for reverse causality.

## Notes

Supplement 1. **eMethods.** Eligibility and Data Management **eTable 1.** Clinical Codes Used to Define Traumatic Brain Injury **eTable 2.** *ICD-10* Codes Used to Define Traumatic Brain Injury **eTable 3.** CPRD Codes Used to Define Motor Neurone Disease/Amyotrophic Lateral Sclerosis **eTable 4.** CPRD Codes Used to Define Neurodegenerative Disease **eTable 5.** *ICD-10* Codes Used to Define Neurodegenerative Disease **eFigure 1.** Flowchart of Participants in CPRD GOLD **eFigure 2.** Flowchart of Participants in CPRD Aurum

Supplement 2.
Data Sharing Statement
