# Supplementary material for: Traumatic Brain Injury and Risk of Amyotrophic Lateral Sclerosis
Source: JAMA Netw Open. 2025 Oct 2;8(10):e2535119. doi: 10.1001/jamanetworkopen.2025.35119 (PMC12492050; doi:10.1001/jamanetworkopen.2025.35119)
Supplement: Supplement 1. — eMethods. Eligibility and Data Management eTable 1. Clinical Codes Used to Define Traumatic Brain Injury eTable 2. ICD-10 Codes Used to Define Traumatic Brain Injury eTable 3. CPRD Codes Used to Define Motor Neurone Disease/Amyotrophic Lateral Sclerosis eTable 4. CPRD Codes Used to Define Neurodegenerative Disease eTable 5. ICD-10 Codes Used to Define Neurodegenerative Disease eFigure 1. Flowchart of Participants in CPRD GOLD eFigure 2. Flowchart of Participants in CPRD Aurum [file jamanetwopen-e2535119-s001.pdf]

Supplementary Online Content

Zhu X, Russell ER, Lyall DM, et al. Traumatic brain injury and risk of amyotrophic lateral sclerosis. *JAMA Netw Open.* 2025;8(10):e2535119. doi:10.1001/jamanetworkopen.2025.35119

eMethods. Eligibility and Data Management

eTable 1. Clinical Codes Used to Define Traumatic Brain Injury

eTable 2. *ICD-10* Codes Used to Define Traumatic Brain Injury

eTable 3. CPRD Codes Used to Define Motor Neurone Disease/Amyotrophic Lateral Sclerosis

eTable 4. CPRD Codes Used to Define Neurodegenerative Disease

eTable 5. *ICD-10* Codes Used to Define Neurodegenerative Disease

eFigure 1. Flowchart of Participants in CPRD GOLD

eFigure 2. Flowchart of Participants in CPRD Aurum

This supplementary material has been provided by the authors to give readers additional information about their work.

© 2025 Zhu X et al. *JAMA Network Open*.

eMethods. Eligibility and Data Management

Individuals were eligible if they met the following criteria: (i) having no history of TBI before 2005; (ii) data labelled as acceptable for research by CPRD based on continuity of follow up and quality of data recording; (iii) having medical records available within the study period (2005-2020); (iv) no Read/SNOMED/EMIS (**eTable 4**) or ICD-10 (**eTable 5**) coded neurodegenerative disease diagnosis at any time before the index date; (v) having been registered in CPRD for at least one year prior to the index date to prevent historical recording of ALS diagnoses at registration; and (vi) no record of death, transfer out or end of data collection before the index date.

For Gold specifically, CPRD assigned an up-to-standard (UTS) date for each practice, which represented ‘the date at which data in the practice is considered to have continuous high-quality data fit for use in research’. Data recorded before the UTS date are available but not of an acceptable research standard. We therefore excluded participants from practices that were not UTS by the index date. Duplication of data within Aurum or between the two datasets was also removed. All eligible participants were then followed up from the index date, until the date of the first ALS incidence, death from any cause, migration from general practice, the end of data collection by the practice, or the end of the study period (31st December 2020), whichever occurred first. The flowchart of participants is provided in **eFigures 1 and 2.** The majority of the N=150 ALS cases were identified within CPRD (89 cases), with additional cases identified in HES (51 cases) and a small number from death certification data (three cases). Six were recorded with ALS in both CPRD and ONS on the same date. Another one ALS diagnosis appeared in CPRD and HES on the same date.

© 2025 Zhu X et al. *JAMA Network Open*.

eTable 1. Clinical Codes Used to Define Traumatic Brain Injury

Database	medcode	readcode	readterm
GOLD	403	S64..13	Head injury
GOLD	3008	S646.00	Head injury
GOLD	6431	14J1.00	H/O: head injury
GOLD	16553	S64..12	Head injury NOS
GOLD	10201	S64..11	Brain injury NOS
GOLD	27632	S643.00	Diffuse brain injury
GOLD	32214	S644.00	Focal brain injury
Aurum	817721000006116	S64-3	Head injury
Aurum	136462012	S646	Head injury
Aurum	383815100000611 2	^ESCTHI383815	HI - Head injury
Aurum	907031000006111	HNG0156	[RFC] Head injury
Aurum	750611000006113	SC20-1	Sequelae of injuries of head
Aurum	251820017	14J1	H/O: head injury
Aurum	454169100000611 6	^ESCTHI454169	History of head injury
Aurum	390461014	S64-2	Head injury NOS
Aurum	390672011	S64-1	Traumatic AND/OR non-traumatic brain injury
Aurum	411234012	SC20z-1	Traumatic brain injury
Aurum	180853100000611 3	EMISNQTR31	Traumatic brain injury
Aurum	802647100000611 0	^ESCTHI802647	History of traumatic brain injury
Aurum	390682012	S643	Diffuse brain injury
Aurum	320930015	S644	Focal brain injury
Aurum	459051012	ESCTMA1	Major head injury
Aurum	439632100000611 2	^ESCTTR43963 2	Traumatic brain injury with no loss of consciousness
Aurum	439630100000611 9	^ESCTTR43963 0	Traumatic brain injury with moderate loss of consciousness
Aurum	738059100000611 4	^ESCTTR73805 9	Traumatic brain injury with loss of consciousness one hour or more
Aurum	439628100000611 8	^ESCTTR43962 8	Traumatic brain injury with loss of consciousness
Aurum	439629100000611 5	^ESCTTR43962 9	Traumatic brain injury with brief loss of consciousness

© 2025 Zhu X et al. *JAMA Network Open*.

eTable 2. *ICD-10* Codes Used to Define Traumatic Brain Injury

**ICD-10 codes**	Description
S06.0	Concussion
S06.1	Traumatic cerebral oedema
S06.2	Diffuse brain injury
S06.3	Focal brain injury
S06.4	Epidural haemorrhage
S06.5	Traumatic subdural haemorrhage
S06.6	Traumatic subarachnoid haemorrhage
S06.7	Intracranial injury with prolonged coma
S06.8	Other intracranial injuries
S06.9	Intracranial injury, unspecified
S07.1	Crushing injury of skull
S07.8	Crushing injury of other parts of head
S07.9	Crushing injury of head, part unspecified
S09.7	Multiple injuries of head
S09.8	Other specified injuries of head
S09.9	Unspecified injury of head

© 2025 Zhu X et al. *JAMA Network Open*.

eTable 3. CPRD Codes Used to Define Motor Neurone Disease/Amyotrophic Lateral Sclerosis

Database	medcode	readcode	readterm
GOLD	20845	F152400	Primary lateral sclerosis
GOLD	36433	F152000	Amyotrophic lateral sclerosis
GOLD	109020	7Q04100	Amyotrophic lateral sclerosis drugs Band 1
GOLD	30987	F152100	Progressive muscular atrophy
GOLD	27377	F152200	Progressive bulbar palsy
GOLD	4796	F152.00	Motor neurone disease
GOLD	20120	F152z00	Motor neurone disease NOS
Aurum	486695013	F152	Motor neurone disease
Aurum	3101721000006110	^ESCTMN310172	MND - Motor neurone disease
Aurum	297130012	F152z	Motor neurone disease NOS
Aurum	908821000006116	HNG0604	[RFC] Motor neurone disease
Aurum	905801000006119	HNG0053	[RFC] Motor neurone disease
Aurum	3101721000006110	^ESCTMN310172	MND - Motor neurone disease
Aurum	3898641000006114	^ESCTBU389864	Bulbar motor neuron disease
Aurum	134744017	F1524	Primary lateral sclerosis
Aurum	142653015	F1520	Amyotrophic lateral sclerosis
Aurum	3898661000006113	^ESCTAL389866	ALS - Amyotrophic lateral sclerosis
Aurum	351861000000110	7Q041	Amyotrophic lateral sclerosis drugs Band 1
Aurum	3381211000006117	^ESCTPB338121	PBP - Progressive bulbar palsy
Aurum	90263012	F1522	Progressive bulbar palsy
Aurum	3547301000006112	^ESCTPR354730	Progressive muscular atrophy of infancy
Aurum	3945261000006119	^ESCTPM394526	PMA - Progressive muscular atrophy
Aurum	3945251000006116	^ESCTPU394525	Pure progressive muscular atrophy
Aurum	147444011	F1521	Progressive muscular atrophy
Aurum	5005371000006112	^ESCTBU500537	Bulbospinal muscular atrophy

© 2025 Zhu X et al. *JAMA Network Open*.

eTable 4. CPRD Codes Used to Define Neurodegenerative Disease

Database	medcode	readcode	readterm
GOLD	8956	F130300	Parkinsonism with orthostatic hypotension
GOLD	14912	F12z.00	Parkinson's disease NOS
GOLD	33544	F121.00	Parkinsonism secondary to drugs
GOLD	4321	F12..00	Parkinson's disease
GOLD	16797	F110000	Alzheimer's disease with early onset
GOLD	32057	F110100	Alzheimer's disease with late onset
GOLD	1917	F110.00	Alzheimer's disease
GOLD	60059	Eu00012	[X]Primary degen dementia, Alzheimer's type, presenile onset
GOLD	61528	Eu00013	[X]Alzheimer's disease type 2
GOLD	25704	Eu00011	[X]Presenile dementia,Alzheimer's type
GOLD	49263	Eu00000	[X]Dementia in Alzheimer's disease with early onset
GOLD	38678	Eu00100	[X]Dementia in Alzheimer's disease with late onset
GOLD	7664	Eu00.00	[X]Dementia in Alzheimer's disease
GOLD	43346	Eu00113	[X]Primary degen dementia of Alzheimer's type, senile onset
GOLD	11379	Eu00112	[X]Senile dementia,Alzheimer's type
GOLD	46762	Eu00111	[X]Alzheimer's disease type 1
GOLD	26421	ZR1K.11	ADAS - Alzheimer's disease assessment scale
GOLD	8195	Eu00z11	[X]Alzheimer's dementia unspec
GOLD	30706	Eu00200	[X]Dementia in Alzheimer's dis, atypical or mixed type
GOLD	59122	Fyu3000	[X]Other Alzheimer's disease
GOLD	29386	Eu00z00	[X]Dementia in Alzheimer's disease, unspecified
GOLD	42602	E001000	Uncomplicated presenile dementia
GOLD	38438	E001z00	Presenile dementia NOS

© 2025 Zhu X et al. *JAMA Network Open*.

GOLD	44674	E002.00	Senile dementia with depressive or paranoid features
GOLD	41185	Eu02400	[X]Dementia in human immunodef virus [HIV] disease
GOLD	43089	E004000	Uncomplicated arteriosclerotic dementia
GOLD	26323	Eu10711	[X]Alcoholic dementia NOS
GOLD	15165	E001.00	Presenile dementia
GOLD	49513	E001100	Presenile dementia with delirium
GOLD	109047	8BPa.00	Antipsychotic drug therapy for dementia
GOLD	6578	Eu01.00	[X]Vascular dementia
GOLD	41089	E002z00	Senile dementia with depressive or paranoid features NOS
GOLD	56912	E004100	Arteriosclerotic dementia with delirium
GOLD	62132	E02y100	Drug-induced dementia
GOLD	26270	Eu02500	[X]Lewy body dementia
GOLD	43292	E004300	Arteriosclerotic dementia with depression
GOLD	37015	E003.00	Senile dementia with delirium
GOLD	104155	1JA2.00	Suspected dementia
GOLD	19477	E004.00	Arteriosclerotic dementia
GOLD	55467	E004200	Arteriosclerotic dementia with paranoia
GOLD	42279	E004z00	Arteriosclerotic dementia NOS
GOLD	9565	Eu01.11	[X]Arteriosclerotic dementia
GOLD	9509	Eu02300	[X]Dementia in Parkinson's disease
GOLD	46488	Eu01000	[X]Vascular dementia of acute onset
GOLD	8634	E004.11	Multi infarct dementia
GOLD	28402	Eu02000	[X]Dementia in Pick's disease
GOLD	107389	38C1300	Assessment of psychotic and behavioural symptoms of dementia
GOLD	1916	E00..11	Senile dementia
GOLD	31016	Eu01300	[X]Mixed cortical and subcortical vascular dementia

© 2025 Zhu X et al. *JAMA Network Open*.

GOLD	55313	Eu01y00	[X]Other vascular dementia
GOLD	53446	Eu04100	[X]Delirium superimposed on dementia
GOLD	48501	Eu02z11	[X] Presenile dementia NOS
GOLD	27759	Eu02z16	[X] Senile dementia, depressed or paranoid type
GOLD	4357	Eu02z14	[X] Senile dementia NOS
GOLD	21887	E002100	Senile dementia with depression
GOLD	54106	Eu02100	[X]Dementia in Creutzfeldt-Jakob disease
GOLD	5931	1461	H/O: dementia
GOLD	55838	Eu01111	[X]Predominantly cortical dementia
GOLD	30032	E001200	Presenile dementia with paranoia
GOLD	34944	Eu02z13	[X] Primary degenerative dementia NOS
GOLD	8934	Eu01200	[X]Subcortical vascular dementia
GOLD	1350	E00..12	Senile/presenile dementia
GOLD	18386	E002000	Senile dementia with paranoia
GOLD	27342	E012.11	Alcoholic dementia NOS
GOLD	37014	Eu02200	[X]Dementia in Huntington's disease
GOLD	54505	E012.00	Other alcoholic dementia
GOLD	4693	Eu02z00	[X] Unspecified dementia
GOLD	27677	E001300	Presenile dementia with depression
GOLD	64267	Eu02y00	[X]Dementia in other specified diseases classif elsewhere
GOLD	19393	Eu01z00	[X]Vascular dementia, unspecified
GOLD	19393	Eu01z00	[X]Vascular dementia, unspecified
GOLD	53697	ZS42212	Lower motor neurone type of dysarthria
GOLD	4796	F152.00	Motor neurone disease
GOLD	20120	F152z00	Motor neurone disease NOS
GOLD	25387	ZS42114	Upper motor neurone type of dysarthria
GOLD	36433	F152000	Amyotrophic lateral sclerosis
GOLD	30987	F152100	Progressive muscular atrophy
GOLD	27377	F152200	Progressive bulbar palsy
GOLD	20845	F152400	Primary lateral sclerosis

© 2025 Zhu X et al. *JAMA Network Open*.

GOLD	109020	7Q04100	Amyotrophic lateral sclerosis drugs Band 1
GOLD	7572	F116.00	Lewy body disease
GOLD	11175	Eu01100	[X]Multi-infarct dementia
GOLD	7323	E000.00	Uncomplicated senile dementia
GOLD	109737	8BM0200	Dementia medication review
GOLD	12710	6AB..00	Dementia annual review
GOLD	25386	E041.00	Dementia in conditions EC
GOLD	12621	Eu02.00	[X]Dementia in other diseases classified elsewhere
GOLD	26323	Eu10711	[X]Alcoholic dementia NOS
Aurum	21256010	E001	Presenile dementia
Aurum	33622011	F123	Postencephalitic parkinsonism
Aurum	45046017	F110	Alzheimer's disease
Aurum	64875013	A94y1	Syphilitic parkinsonism
Aurum	81717011	F12	Parkinson's disease
Aurum	142653015	F1520	Amyotrophic lateral sclerosis
Aurum	251625013	1461	H/O: dementia
Aurum	294635013	E000	Uncomplicated senile dementia
Aurum	294638010	E0011	Presenile dementia with delirium
Aurum	294641018	E0012	Presenile dementia with paranoia
Aurum	294642013	E0013	Presenile dementia with depression
Aurum	294643015	E001z	Presenile dementia NOS
Aurum	294644014	E002	Senile dementia with depressive or paranoid features
Aurum	294645010	E0020	Senile dementia with paranoia
Aurum	294656010	E004z	Arteriosclerotic dementia NOS
Aurum	294688019	E02y1	Drug-induced dementia
Aurum	294718018	E041	Dementia in conditions EC
Aurum	295668011	Eu00	[X]Dementia in Alzheimer's disease
Aurum	295671015	Eu002	[X]Dementia in Alzheimer's dis, atypical or mixed type

© 2025 Zhu X et al. *JAMA Network Open*.

Aurum	295672010	Eu00z	[X]Dementia in Alzheimer's disease, unspecified
Aurum	295681016	Eu01z	[X]Vascular dementia, unspecified
Aurum	295686014	Eu021	[X]Dementia in Creutzfeldt-Jakob disease
Aurum	295687017	Eu022	[X]Dementia in Huntington's disease
Aurum	295688010	Eu023	[X]Dementia in Parkinson's disease
Aurum	295714013	Eu041	[X]Delirium superimposed on dementia
Aurum	297037012	F12z	Parkinson's disease NOS
Aurum	297043014	F1303	Parkinsonism with orthostatic hypotension
Aurum	297130012	F152z	Motor neurone disease NOS
Aurum	299312017	Fyu20	[X]Other drug-induced secondary parkinsonism
Aurum	299313010	Fyu21	[X]Other secondary parkinsonism
Aurum	299314016	Fyu22	[X]Parkinsonism in diseases classified elsewhere
Aurum	299321016	Fyu29	[X]Secondary parkinsonism, unspecified
Aurum	299323018	Fyu2B	[X]Secondary parkinsonism due to other external agents
Aurum	299325013	Fyu30	[X]Other Alzheimer's disease
Aurum	324219016	SL6yz	Antiparkinsonian drug poisoning NOS
Aurum	345121017	F124	Vascular parkinsonism
Aurum	346929012	E012-1	Alcoholic dementia NOS
Aurum	486695013	F152	Motor neurone disease
Aurum	499946014	F1100	Alzheimer's disease with early onset
Aurum	500317011	F1101	Alzheimer's disease with late onset
Aurum	2841061014	F1741	Multiple system atrophy, Parkinson variant
Aurum	148381000006115	E00-2	Senile/presenile dementia
Aurum	151951000006115	F12W	Secondary parkinsonism due to other external agents
Aurum	151961000006118	F12X	Secondary parkinsonism, unspecified
Aurum	244311000006117	F121	Parkinsonism secondary to drugs
Aurum	299641000000112	Eu025	[X]Lewy body dementia
Aurum	359081000006118	Eu02z-1	[X] Presenile dementia NOS
Aurum	359101000006114	Eu02z-3	[X] Primary degenerative dementia NOS

© 2025 Zhu X et al. *JAMA Network Open*.

Aurum	35914100006111	Eu02z-4	[X] Senile dementia NOS
Aurum	35915100006113	Eu02z-6	[X] Senile dementia, depressed or paranoid type
Aurum	35924100006119	Eu02z	[X] Unspecified dementia
Aurum	36294100006113	Eu107-1	[X]Alcoholic dementia NOS
Aurum	36302100006113	Eu00z-1	[X]Alzheimer's dementia unspec
Aurum	36303100006111	Eu001-1	[X]Alzheimer's disease type 1
Aurum	36304100006118	Eu000-3	[X]Alzheimer's disease type 2
Aurum	36379100006112	Eu01-1	[X]Arteriosclerotic dementia
Aurum	37653100006119	Eu000	[X]Dementia in Alzheimer's disease with early onset
Aurum	37654100006112	Eu001	[X]Dementia in Alzheimer's disease with late onset
Aurum	37657100006116	Eu024	[X]Dementia in human immunodef virus [HIV] disease
Aurum	39857100006112	Eu013	[X]Mixed cortical and subcortical vascular dementia
Aurum	39903100006111	Eu011	[X]Multi-infarct dementia
Aurum	42322100006117	Eu011-1	[X]Predominantly cortical dementia
Aurum	42335100006115	Eu000-1	[X]Presenile dementia,Alzheimer's type
Aurum	42338100006111	Eu001-3	[X]Primary degen dementia of Alzheimer's type, senile onset
Aurum	42339100006114	Eu000-2	[X]Primary degen dementia, Alzheimer's type, presenile onset
Aurum	42590100006116	Eu001-2	[X]Senile dementia,Alzheimer's type
Aurum	42820100006119	Eu012	[X]Subcortical vascular dementia
Aurum	43168100006117	Eu01	[X]Vascular dementia
Aurum	43169100006119	Eu010	[X]Vascular dementia of acute onset
Aurum	63049100006110	F121-1	Drug induced parkinsonism
Aurum	63668100000117	F11x9	Cerebral degeneration in Parkinson's disease
Aurum	66023100006117	F13-1	Extrapyramidal disease excluding Parkinson's disease
Aurum	69616100006115	E004-1	Multi infarct dementia

© 2025 Zhu X et al. *JAMA Network Open*.

Aurum	745381000006119	F116	Lewy body disease
Aurum	882171000006115	E00-97	Dementia
Aurum	882201000006116	E000-99	Senile dementia - simple type
Aurum	882211000006118	E003-99	Senile dementia-acute confused
Aurum	882961000006112	F121-99	Secondary Parkinsonism - drugs
Aurum	905791000006115	HNG0062	[RFC] Alzheimer's disease
Aurum	905801000006119	HNG0053	[RFC] Motor neurone disease
Aurum	905821000006112	HNG0054	[RFC] Parkinson's disease
Aurum	908821000006116	HNG0604	[RFC] Motor neurone disease
Aurum	909021000006117	HNG0633	[RFC] Parkinson's disease
Aurum	914921000006117	EMISNQDV1	[D] Vascular dementia
Aurum	914931000006119	EMISNQDD1	[D] Dementia with Lewy bodies
Aurum	914951000006114	EMISNQDD3	[D] Dementia in Alzheimer's disease
Aurum	939491000006118	HNGNQRF130	[RFC] Dementia
Aurum	1148731000000112	147F	History of Parkinson's disease
Aurum	1576281000006119	EGTON2EGTONAL1	Cause of Death- Alzheimer'S Disease
Aurum	1776901000006117	EMISNQRE248	Reason for referral: Parkinsons Disease
Aurum	1823871000006112	EMISNQDE35	Dementia confirmed
Aurum	1949641000006113	EMISNQDE69	Dementia stage at diagnosis - mid (moderate)
Aurum	1949651000006110	EMISNQDE70	Dementia stage at diagnosis - late (severe)
Aurum	1971401000006111	EMISICD10|F0000	Dementia in Alzheimer's disease with early onset, without additional symptoms
Aurum	1971541000006114	EMISICD10|F0001	Dementia in Alzheimer's disease with early onset, other symptoms, predominantly delusional
Aurum	1971771000006112	EMISICD10|F0002	Dementia in Alzheimer's disease with early onset, other symptoms, predominantly hallucinatory
Aurum	1972021000006119	EMISICD10|F03X0	Unspecified dementia, without additional symptoms
Aurum	1972081000006115	EMISICD10|F03X4	Unspecified dementia, other mixed symptoms
Aurum	1972131000006115	EMISICD10|F0003	Dementia in Alzheimer's disease with early onset, other symptoms, predominantly depressive

© 2025 Zhu X et al. *JAMA Network Open*.

Aurum	1972141000006113	EMISICD10|F0004	Dementia in Alzheimer's disease with early onset, other mixed symptoms
Aurum	1972171000006117	EMISICD10|F0010	Dementia in Alzheimer's disease with late onset, without additional symptoms
Aurum	1972181000006119	EMISICD10|F0011	Dementia in Alzheimer's disease with late onset, other symptoms, predominantly delusional
Aurum	1972191000006116	EMISICD10|F0012	Dementia in Alzheimer's disease with late onset, other symptoms, predominantly hallucinatory
Aurum	1972201000006118	EMISICD10|F0013	Dementia in Alzheimer's disease with late onset, other symptoms, predominantly depressive
Aurum	1972211000006115	EMISICD10|F0014	Dementia in Alzheimer's disease with late onset, other mixed symptoms
Aurum	1972231000006114	EMISICD10|F0020	Dementia in Alzheimer's dis, atypical or mixed type, without additional symptoms
Aurum	1972251000006119	EMISICD10|F0021	Dementia in Alzheimer's dis, atypical or mixed type, other symptoms, predominantly delusional
Aurum	1972291000006113	EMISICD10|F0022	Dementia in Alzheimer's dis, atypical or mixed type, other symptoms, predominantly hallucinatory
Aurum	1972311000006112	EMISICD10|F0023	Dementia in Alzheimer's dis, atypical or mixed type, other symptoms, predominantly depressive
Aurum	1972341000006111	EMISICD10|F0024	Dementia in Alzheimer's dis, atypical or mixed type, other mixed symptoms
Aurum	1972371000006115	EMISICD10|F0090	Dementia in Alzheimer's disease, unspecified, without additional symptoms
Aurum	1972401000006117	EMISICD10|F0091	Dementia in Alzheimer's disease, unspecified, other symptoms, predominantly delusional
Aurum	1972421000006110	EMISICD10|F0092	Dementia in Alzheimer's disease, unspecified, other symptoms, predominantly hallucinatory
Aurum	1972431000006113	EMISICD10|F1273	Mental & behav dis due to cannabinoids: resid & late-onset psychot dis, dementia
Aurum	1972451000006118	EMISICD10|F0093	Dementia in Alzheimer's disease, unspecified, other symptoms, predominantly depressive

© 2025 Zhu X et al. *JAMA Network Open*.

Aurum	1972471000006111	EMISICD10|F0094	Dementia in Alzheimer's disease, unspecified, other mixed symptoms
Aurum	1972481000006114	EMISICD10|F0100	Vascular dementia of acute onset, without additional symptoms
Aurum	1972501000006116	EMISICD10|F0101	Vascular dementia of acute onset, other symptoms, predominantly delusional
Aurum	1972621000006113	EMISICD10|F0111	Multi-infarct dementia, other symptoms, predominantly delusional
Aurum	1972641000006118	EMISICD10|F0112	Multi-infarct dementia, other symptoms, predominantly hallucinatory
Aurum	1972661000006119	EMISICD10|F0113	Multi-infarct dementia, other symptoms, predominantly depressive
Aurum	1972681000006112	EMISICD10|F0114	Multi-infarct dementia, other mixed symptoms
Aurum	1972711000006113	EMISICD10|F0120	Subcortical vascular dementia, without additional symptoms
Aurum	1972731000006119	EMISICD10|F0121	Subcortical vascular dementia, other symptoms, predominantly delusional
Aurum	1972771000006116	EMISICD10|F0123	Subcortical vascular dementia, other symptoms, predominantly depressive
Aurum	1973221000006118	EMISICD10|F0180	Other vascular dementia, without additional symptoms
Aurum	1973271000006117	EMISICD10|F0181	Other vascular dementia, other symptoms, predominantly delusional
Aurum	1973381000006112	EMISICD10|F0183	Other vascular dementia, other symptoms, predominantly depressive
Aurum	1973401000006112	EMISICD10|F0184	Other vascular dementia, other mixed symptoms
Aurum	1973551000006112	EMISICD10|F0193	Vascular dementia, unspecified, other symptoms, predominantly depressive
Aurum	1976831000006111	EMISICD10|F0194	Vascular dementia, unspecified, other mixed symptoms
Aurum	2127581000000115	1JA2	Suspected dementia
Aurum	2502971000006115	^ESCTDE250297	Dementia associated with alcoholism
Aurum	2502981000006117	^ESCTAL250298	Alcohol-induced persisting dementia

© 2025 Zhu X et al. *JAMA Network Open*.

Aurum	2566251000006117	^ESCTPA256625	Parkinsonism caused by drug
Aurum	2748441000006111	^ESCTSD274844	SD - Senile dementia
Aurum	2931231000006118	^ESCTAD293123	AD - Alzheimer's disease
Aurum	2931241000006111	^ESCTAL293124	Alzheimer disease
Aurum	2931251000006113	^ESCTAL293125	Alzheimer dementia
Aurum	2966081000006114	^ESCTNU296608	Nuchal dystonia-dementia syndrome
Aurum	3028151000006111	^ESCTPA302815	Parkinsonism
Aurum	3028161000006113	^ESCTDI302816	Disorders presenting primarily with parkinsonism
Aurum	3101701000006117	^ESCTMO310170	Motor neuron disease
Aurum	3101721000006110	^ESCTMN310172	MND - Motor neurone disease
Aurum	3293631000006118	^ESCTID329363	Idiopathic Parkinson's disease
Aurum	3293641000006111	^ESCTPA329364	Parkinson disease
Aurum	3293651000006113	^ESCTPD329365	PD - Parkinson's disease
Aurum	3293661000006110	^ESCTPA329366	Parkinsons disease
Aurum	3293671000006115	^ESCTPR329367	Primary Parkinsonism
Aurum	3293681000006117	^ESCTID329368	Idiopathic Parkinsonism
Aurum	3341641000006113	^ESCTDE334164	Dementia paralytica
Aurum	3341701000006116	^ESCTPA334170	Paralytic dementia
Aurum	3350441000006115	^ESCTOR335044	Organic dementia
Aurum	3414231000006117	^ESCTMI341423	MID - Multi-infarct dementia
Aurum	3414251000006112	^ESCTVA341425	VAD - Vascular dementia
Aurum	3414261000006114	^ESCTMU341426	Multi infarct dementia
Aurum	3802621000006119	^ESCTLE380262	Lewy body variant of Alzheimer's disease
Aurum	3802631000006116	^ESCTSD380263	SDLT - Senile dementia of the Lewy body type
Aurum	3802641000006114	^ESCTLB380264	LBD - Lewy body disease
Aurum	3802651000006111	^ESCTDE380265	Dementia of the Lewy body type
Aurum	3802661000006113	^ESCTDL380266	DLBD - Diffuse Lewy body disease
Aurum	3802671000006118	^ESCTCO380267	Cortical Lewy body disease
Aurum	3802681000006115	^ESCTCL380268	CLBD - Cortical Lewy body disease
Aurum	3898641000006114	^ESCTBU389864	Bulbar motor neuron disease
Aurum	3898661000006113	^ESCTAL389866	ALS - Amyotrophic lateral sclerosis

© 2025 Zhu X et al. *JAMA Network Open*.

Aurum	3964611000006111	^ESCTBI396461	Binswanger's dementia
Aurum	3964661000006114	^ESCTSU396466	Subcortical atherosclerotic dementia
Aurum	4539871000006116	^ESCTHI453987	History of dementia
Aurum	5005921000006112	^ESCTSE500592	Secondary Parkinson's disease
Aurum	5005931000006110	^ESCTSE500593	Secondary Parkinson disease
Aurum	6897211000006117	^ESCTPR689721	Primary degenerative dementia of the Alzheimer type, presenile onset
Aurum	6897221000006113	^ESCTPR689722	Primary degenerative dementia of the Alzheimer type, early onset
Aurum	6897241000006118	^ESCTDE689724	Dementia of the Alzheimers type with early onset
Aurum	6897251000006116	^ESCTPR689725	Presenile dementia, Alzheimer's type
Aurum	6897271000006114	^ESCTDE689727	Dementia in Alzheimer's disease - type 2
Aurum	6900181000006114	^ESCTPR690018	Primary degenerative dementia of the Alzheimer type, senile onset
Aurum	6900191000006112	^ESCTPR690019	Primary degenerative dementia of the Alzheimer type, late onset
Aurum	6900201000006110	^ESCTDE690020	Dementia of the Alzheimers type, late onset
Aurum	6900221000006117	^ESCTSD690022	SDAT - Senile dementia, Alzheimer's type
Aurum	6900241000006112	^ESCTDE690024	Dementia in Alzheimer's disease - type 1
Aurum	6973421000006116	^ESCTDE697342	Dementia associated with AIDS
Aurum	6973431000006118	^ESCTAC697343	Acquired immune deficiency syndrome-related dementia
Aurum	6973471000006115	^ESCTDE697347	Dementia associated with acquired immunodeficiency syndrome
Aurum	7043651000006119	^ESCTDE704365	Dementia associated with Parkinson's Disease
Aurum	7043661000006117	^ESCTDE704366	Dementia associated with Parkinson Disease
Aurum	7103601000006116	^ESCTDE710360	Dementia due to Creutzfeldt-Jakob disease
Aurum	7263011000006112	^ESCTDE726301	Dementia due to Huntington disease
Aurum	7263021000006116	^ESCTDE726302	Dementia due to Huntingtons disease
Aurum	7289421000006111	^ESCTMU728942	Multiple system atrophy, Parkinson's variant
Aurum	8009521000006114	^ESCTDE800952	Dementia due to Picks disease
Aurum	8009531000006112	^ESCTDE800953	Dementia due to Pick disease

© 2025 Zhu X et al. *JAMA Network Open*.

Aurum	8009541000006119	^ESCTDE800954	Dementia co-occurrent and due to Pick's disease
Aurum	8193781000006112	^ESCTHI819378	History of Parkinson disease
Aurum	294646011	E0021	Senile dementia with depression
Aurum	294655014	E0043	Arteriosclerotic dementia with depression
Aurum	486695013	F152	Motor neurone disease
Aurum	3101721000006110	^ESCTMN310172	MND - Motor neurone disease
Aurum	297130012	F152z	Motor neurone disease NOS
Aurum	908821000006116	HNG0604	[RFC] Motor neurone disease
Aurum	905801000006119	HNG0053	[RFC] Motor neurone disease
Aurum	3101721000006110	^ESCTMN310172	MND - Motor neurone disease
Aurum	3898641000006114	^ESCTBU389864	Bulbar motor neuron disease
Aurum	134744017	F1524	Primary lateral sclerosis
Aurum	142653015	F1520	Amyotrophic lateral sclerosis
Aurum	3898661000006113	^ESCTAL389866	ALS - Amyotrophic lateral sclerosis
Aurum	351861000000110	7Q041	Amyotrophic lateral sclerosis drugs Band 1
Aurum	3381211000006117	^ESCTPB338121	PBP - Progressive bulbar palsy
Aurum	90263012	F1522	Progressive bulbar palsy
Aurum	3547301000006112	^ESCTPR354730	Progressive muscular atrophy of infancy
Aurum	3945261000006119	^ESCTPM394526	PMA - Progressive muscular atrophy
Aurum	3945251000006116	^ESCTPU394525	Pure progressive muscular atrophy
Aurum	147444011	F1521	Progressive muscular atrophy
Aurum	5005371000006112	^ESCTBU500537	Bulbospinal muscular atrophy

© 2025 Zhu X et al. *JAMA Network Open*.

eTable 5. *ICD-10* Codes Used to Define Neurodegenerative Disease

ICD-10 codes	Description
F01	Vascular dementia
F01.0	Vascular dementia of acute onset
F01.1	Multi-infarct dementia
F01.2	Subcortical vascular dementia
F01.3	Mixed cortical and subcortical vascular dementia
F01.8	Other vascular dementia
F01.9	Vascular dementia, unspecified
F02	*Dementia in other diseases classified elsewhere
F02.0	*Dementia in Pick disease (G31.0†)
F02.1	*Dementia in Creutzfeldt-Jakob disease (A81.0†)
F02.2	*Dementia in Huntington disease (G10†)
F02.3	*Dementia in Parkinson disease (G20†)
F02.4	*Dementia in human immunodeficiency virus [HIV] disease (B22.0†)
F02.8	*Dementia in other specified diseases classified elsewhere
F03	Unspecified dementia
G12.2	Motor neuron disease/ Amyotrophic Lateral Sclerosis
G20	Parkinson disease
G21	Secondary parkinsonism
G21.0	Malignant neuroleptic syndrome
G21.1	Other drug-induced secondary parkinsonism
G21.2	Secondary parkinsonism due to other external agents
G21.3	Postencephalitic parkinsonism
G21.4	Vascular parkinsonism
G21.8	Other secondary parkinsonism
G21.9	Secondary parkinsonism, unspecified
G23.1	Progressive supranuclear ophthalmoplegia [Steele-Richardson-Olszewski]
G23.2	Multiple system atrophy, parkinsonian type [MSA-P]
G23.3	Multiple system atrophy, cerebellar type [MSA-C]
G23.8	Other specified degenerative diseases of basal ganglia
G23.9	Degenerative disease of basal ganglia, unspecified
G30	Alzheimer disease
G30.0	Alzheimer disease with early onset
G30.1	Alzheimer disease with late onset
G30.8	Other Alzheimer disease
G30.9	Alzheimer disease, unspecified
G31	Other degenerative diseases of nervous system, not elsewhere classified
G31.0	Circumscribed brain atrophy
G31.1	Senile degeneration of brain, not elsewhere classified
G31.2	Degeneration of nervous system due to alcohol

© 2025 Zhu X et al. *JAMA Network Open*.

G31.8	Other specified degenerative diseases of nervous system
G31.9	Degenerative disease of nervous system, unspecified

© 2025 Zhu X et al. *JAMA Network Open*.

eFigure 1. Flowchart of Participants in CPRD GOLD

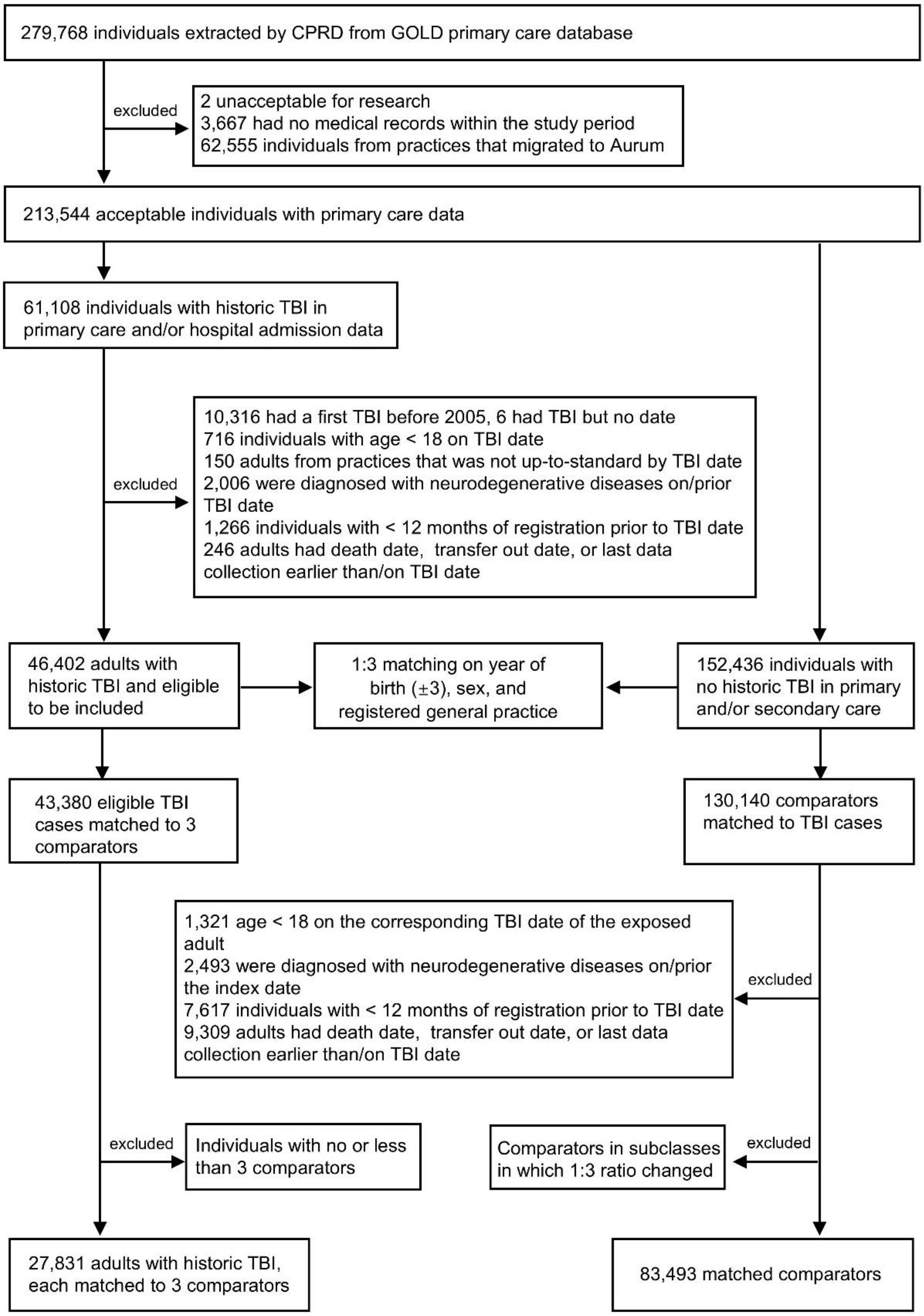

© 2025 Zhu X et al. *JAMA Network Open*.

eFigure 2. Flowchart of Participants in CPRD Aurum

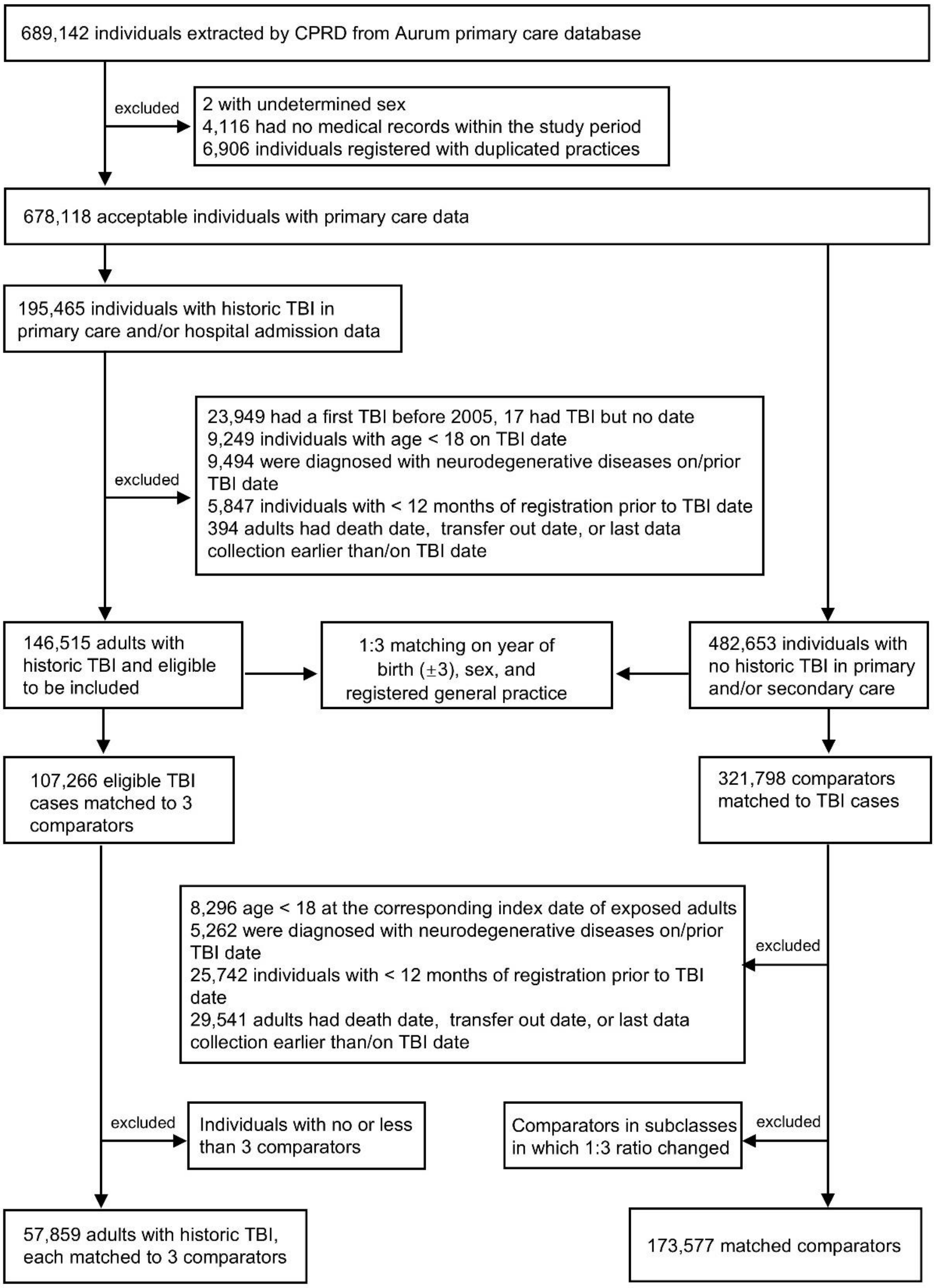

© 2025 Zhu X et al. *JAMA Network Open*.
