# Supplementary material for: Traumatic Brain Injury and Risk of Amyotrophic Lateral Sclerosis
Source: JAMA Netw Open. 2025 Oct 2;8(10):e2535119. doi: 10.1001/jamanetworkopen.2025.35119 (PMC12492050; doi:10.1001/jamanetworkopen.2025.35119)
Supplement: Supplement 2. — Data Sharing Statement [file jamanetwopen-e2535119-s002.pdf]

Data Sharing Statement

Zhu. Traumatic Brain Injury and Risk of Amyotrophic Lateral Sclerosis. *JAMA Netw Open*. Published October 02, 2025. doi:10.1001/jamanetworkopen.2025.35119

Data
Data available: No

Additional Information
Explanation for why data not available: Data are available from CPRD upon application.
